# Current Status of Tivozanib in the Treatment of Patients With Advanced Renal Cell Carcinoma

**DOI:** 10.7759/cureus.35675

**Published:** 2023-03-02

**Authors:** Minas Sakellakis, Roubini Zakopoulou

**Affiliations:** 1 Medical Oncology, Hellenic Genitourinary Cancer Group, Athens, GRC; 2 2nd Propaedeutic Department of Internal Medicine, National and Kapodistrian University of Athens, Attikon University Hospital, Athens, GRC

**Keywords:** renal cell carcinoma, safety, efficacy, sorafenib, tivozanib

## Abstract

The introduction of tyrosine kinase inhibitors (TKIs) against vascular endothelial growth factor receptors (VEGFRs) has transformed the therapeutic landscape for patients with advanced renal cell carcinoma (RCC). However, dose reductions and interruptions are frequently needed due to limited toxicity, mostly from off-target effects. Tivozanib is a potent, selective VEGFR TKI with weak off-target effects. TIVO-1 and TIVO-3 were randomized controlled phase 3 trials that investigated the efficacy and safety of tivozanib versus sorafenib as initial targeted therapy and after failing two previous lines (including targeted therapy), respectively. Tivozanib did not confer any survival advantage, but it significantly increased progression-free survival, response rates, and the duration of responses with a superior safety profile. Although results from subgroup analysis need to be interpreted cautiously, tivozanib demonstrated superiority after two previous lines of VEGFR TKIs or after axitinib, another selective VEGFR inhibitor. Tivozanib also demonstrated durable activity after therapy with an immune-checkpoint inhibitor, while an ongoing study investigating the combination of tivozanib/nivolumab has shown promising preliminary results regarding efficacy and safety. In conclusion, tivozanib was recently added to our therapeutic armamentarium against advanced RCC. Ongoing rational therapeutic combinations of tivozanib will determine the optimal setting in which the maximum benefit can be derived.

## Introduction and background

The American Cancer Society recently estimated that about 79,000 new cases (50,290 men and 28,710 women) of kidney cancer will be diagnosed in the United States during 2022, while 13,920 patients (8,960 men and 4,960 women) will die from the disease [1]. Consistent risk factors include smoking, hypertension, obesity, and chronic kidney dysfunction [2]. The majority of patients with kidney cancer are diagnosed with clear cell carcinoma, which is frequently characterized by the inactivation of the von Hippel-Lindau gene. This results in downstream upregulation of hypoxia-inducible factors 1 alpha and 2 alpha with subsequent pro-tumorigenic gene upregulation, which in turn affects tumor angiogenesis and proliferation [3]. The introduction of tyrosine kinase inhibitors (TKIs) that target neo-angiogenesis by inhibiting vascular endothelial growth factor receptors 1, 2, and 3 (VEGFRs) has transformed the therapeutic landscape in patients with metastatic clear cell, as well as non-clear cell, renal carcinoma. Apart from VEGFRs, older TKIs also bind to other targets, including platelet-derived growth factors (PDGFRs) alpha and beta, c-kit, RET, fms-like tyrosine kinase 3 (FLT3), AXL, Met, fibroblast growth factor receptors (FGFR), tropomyosin receptor kinase B (TRKB), and others [4-6]. Hence, they pose several off-target effects.

Not only are TKIs alone associated with improved clinical outcomes in different treatment lines, but TKIs can also be successfully used in combination with other classes of therapeutic agents, such as immune checkpoint inhibitors (ICIs) [5,6]. Unfortunately, the use of VEGFR TKIs is limited by their considerable toxicity [7,8]. Real-world patients with metastatic kidney cancer, especially after several lines of treatment, are often frail with poor performance status, and they need to take TKIs for long periods of time [9]. As a result, dose reductions, treatment interruptions, or even treatment discontinuation occur frequently [7-9]. Therefore, the potential introduction of novel VEGFR TKIs into clinical practice with similar efficacy and a more favorable toxicity profile would constitute a major advancement. This would not only offer greater flexibility to clinicians to combine VEGFR TKIs with other classes of medications but would also increase the patient's quality of life, especially in the advanced stages of the disease.

## Review

Tivozanib: mechanism of action and dosing schedule

Tivozanib (Fotivda), formerly known as AV-951 or KRN-951, is an oral, potent, selective VEGFR TKI with a weak off-target effect [10]. The inhibitory effect on VEGFRs is stronger compared to other previously used TKIs in metastatic renal-cell carcinoma (mRCC) [11]. However, tivozanib can also inhibit c-kit, which is eight times less sensitive to tivozanib compared to VEGFR 1, 2, and 3. At 10 times higher concentrations, tivozanib can also exert inhibitory effects on PDGFR beta [12]. The recommended dose is 1340 µg once daily for 21 days, followed by a seven-day rest, to comprise a four-week treatment cycle. The medication is administered until disease progression or unacceptable toxicity. Undesirable side effects may lead to temporary treatment interruptions or dose reductions. Food does not affect overall exposure, hence tivozanib can be administered with or without food [12].

Pharmacokinetics and drug metabolism

Peak serum concentrations are achieved after approximately 2 to 24 hours following oral administration of the recommended dose. Half-life is approximately four days. Accumulation of the drug at steady-state is six to seven times higher compared to a single-dose exposure. There are no time-dependent changes in pharmacokinetics, while acute and chronic dosing show similar clearance [12]. More than 99% of the drug is bound to plasma components, especially albumin. Studies suggest that tivozanib is metabolized by cytochrome P450 3A4 (CYP3A4) and CYP1A1, while it can also undergo uridine 5'-diphospho-glucuronosyltransferase (UGT)-mediated biotransformation. There are no major circulating metabolites, but a small amount of the drug (around 12%) can be excreted in the urine in the form of metabolites. The unchanged drug is primarily excreted in the feces [12]. The drug pharmacokinetics are not affected by age, gender, or race. There are no data on the safety and efficacy of the drug in patients under the age of 18. No dose adjustments are needed for patients over 65, patients with mild and moderate renal impairment, and patients with mild hepatic impairment. However, patients with any stage of hepatic dysfunction should be closely monitored. Patients with moderate hepatic disease should be treated with one 1340-µg capsule of tivozanib every other day. Patients with severe hepatic dysfunction should not receive tivozanib, as well as patients with severe renal dysfunction, due to the lack of robust data in this population. Tivozanib is also not recommended in patients with childbearing potential or pregnancy [12]. Tivozanib-induced hypertension (including severe persistent hypertension) has been reported in clinical studies; hence, blood pressure needs to be well controlled before tivozanib initiation. The patients should be closely monitored for hypertension and treated appropriately. Proteinuria has also been reported in clinical studies. All patients need to be monitored for proteinuria before initiation of tivozanib and throughout the treatment periodically. Although grade 2 or 3 proteinuria can be managed with dose reductions or temporary treatment interruptions, grade 4 (nephrotic syndrome) requires treatment discontinuation. Tivozanib should also be used with caution in patients with heart failure or at risk for thromboembolic or bleeding events [12].

The TIVO-1 trial

TIVO-1 was the first open-label, randomized, controlled, phase III study that attempted to investigate the efficacy of tivozanib in advanced renal-cell carcinoma (RCC) [13]. Eligible patients had RCC with a clear cell component, who underwent prior nephrectomy and had recurrent or metastatic disease. The patients were only allowed to receive one or fewer prior systemic treatments (immunotherapy, hormonal therapy, and chemotherapy) for metastatic RCC or in the adjuvant setting if the disease recurred within six months of treatment completion. Patients who received prior VEGF-targeted therapy or prior mammalian target of rapamycin-targeted therapy were excluded. Patients were randomized 1:1 to receive either tivozanib orally at 1.5 mg once daily for three weeks followed by one week off, or sorafenib orally at 400 mg twice a day continuously, as initial targeted therapy. The patients continued treatment until disease progression, unacceptable toxicity, or death [13]. Sorafenib is an agent with proven efficacy in advanced treatment-resistant RCC [14]. Patients who were randomly assigned to sorafenib were allowed to subsequently receive tivozanib in a separate clinical trial [15]. Overall, 517 patients were randomly assigned (n=260 for tivozanib and n=257 for sorafenib), and the baseline patient characteristics were well balanced between the two arms. Based on an independent radiology review, progression-free survival for tivozanib was 11.9 months versus 9.1 months for sorafenib [hazard ratio (HR)=0.797; 95% CI, 0.639 to 0.993; p=0.042] (Table 1). Prespecified subgroup analysis based on baseline characteristics showed a consistent progression-free survival (PFS) advantage for tivozanib over sorafenib. The overall response rate (ORR) was also statistically higher for tivozanib (33.1% vs. 23.3%, p=0.014) [13].

**Table 1:** Efficacy of tivozanib in patients with metastatic renal cell carcinoma Progression-free survival, best responses according to RECIST v1.1, duration of responses, and overall survival in patients who participated in the TIVO-1 and TIVO-3 trials and received either tivozanib or sorafenib. Overall survival results in the TIVO-3 trial have shown inconsistent results at various time points and this outcome is still under evaluation. Abbreviations: *Patients had measurable disease at baseline, NR: not reached, RECIST v1.1: Response Evaluation Criteria in Solid Tumors version 1.1, n/a: not applicable.

	TIVO-1		TIVO-3	
	Tivozanib (n=260)*	Sorafenib (n=257)*	Tivozanib (n=175)*	Sorafenib (n=175)*
	No %	No %	No %	No %
Progression-free survival	11.9 months	9.1 months	5.6 months	3.9 months
Complete response	3 (1.2%)	2 (0.8%)	0	0
Partial response	83 (31.9%)	58 (22.6%)	31 (18%)	14 (8%)
Stable disease	134 (51.5%)	168 (65.4%)	94 (55%)	99 (57%)
Progressive disease	34 (13.1%)	19 (7.4%)	37 (22%)	32 (18%)
Objective response	86 (33.1%)	60 (23.3%)	31 (18%)	14 (8%)
Not evaluable	6 (2.3%)	10 (3.9%)	10 (6%)	30 (17%)
Duration of response	n/a	n/a	NR	5.7 months
Overall survival	28.8 months	29.3 months	n/a	n/a

Surprisingly, overall survival analysis showed that patients on tivozanib had a trend toward lower overall survival compared to patients on sorafenib, although the result did not reach statistical significance (28.8 months vs. 29.3 months; HR, 1.245; 95% CI, 0.954 to 1.624; p=0.105) [13]. However, in the sorafenib arm, 63% of patients received subsequent-line targeted therapy, compared to 13% in the tivozanib arm. Almost all patients in the sorafenib arm who received subsequent targeted therapy received tivozanib [13,15]. Patients from Central or Eastern Europe were far less likely to receive subsequent therapy if they were randomized to tivozanib compared to sorafenib because the majority of patients in the latter group received tivozanib in the separate companion protocol. Interestingly, in the stratum of patients from Western Europe/North America, a trend in overall survival (OS) favoring the tivozanib arm was observed [13,15]. In the post-sorafenib setting, tivozanib demonstrated a median PFS of 11 months and a median OS of 21.6 months [15]. In light of the inconclusive results and the several limitations of the study, which included the absence of blinding, an inappropriate comparator, a one-way crossover, and a lack of internal consistency in the primary endpoint result, the Food and Drug Administration (FDA) rejected the approval of tivozanib at the time [16]. However, the European Medicines Agency (EMA) did approve tivozanib in this setting, based on the positive results of TIVO-1 regarding the primary endpoint of the study [16].

The TIVO-3 trial

In order to address the unanswered questions that arose from the TIVO-1 trial, the TIVO-3 trial was subsequently conducted [17]. TIVO-3 was an open-label, randomized, controlled phase III trial that compared the efficacy and safety of tivozanib with those of sorafenib in the third- or fourth-line setting in metastatic RCC. Overall, 350 patients with metastatic RCC (with a clear cell component) who had previously received two to three previous systemic regimens, at least one of which was a VEGFR TKI apart from tivozanib and sorafenib, were enrolled in the study. In the intention-to-treat (ITT) population, 175 patients received tivozanib, while 175 patients received sorafenib (Table 1). Baseline patient characteristics were well balanced between the two arms. The median PFS for the tivozanib arm was 5.6 months (95% CI 5.29-7.33) versus 3.9 months (95% CI 3.71-5.55) for the sorafenib arm (HR 0.73, 95% CI 0.56-0.94; p=0.016) [17]. One and two-year PFS were 28% and 18%, respectively, for tivozanib versus sorafenib 11% and 5%, respectively. In patients with favorable International Metastatic RCC Database Consortium (IMDC) risk, the median PFS for tivozanib and sorafenib was 11.1 and 6 months, respectively. In patients with intermediate IMDC risk, the median PFS for tivozanib and sorafenib was 5.6 and 5.5 months, while for patients with poor IMDC risk median PFS was 2.1 versus 3.7 months, favoring the sorafenib arm. Although the best response in both arms was a partial response, the overall response rate (ORR) was also higher in the tivozanib arm (p=0.017) [17].

At the conclusion of the report (33 months was the median duration of the study), 20 patients in the tivozanib arm remained disease-free, while only 2 patients in the sorafenib arm remained disease-free. The duration of response was also higher in the tivozanib arm (not reached versus 5.7 months). A longer follow-up analysis showed that three-year PFS was five times higher with tivozanib compared to sorafenib [18]. Patients who previously received treatment with immune checkpoint inhibitors showed a median PFS of 7.3 months with tivozanib compared to 5.1 months with sorafenib. Interestingly, one-year and two-year PFS with tivozanib in these patients were 37% and 28%, respectively, versus 5% and 0% with sorafenib [17].

The median overall survival was not significantly different after a mean follow-up of 17.9 months (16.4 months versus 19.7 months for tivozanib and sorafenib, respectively; HR 0.99, 95% CI 0.76-1.29; p=0.95) [17]. A subsequent OS analysis after a mean follow-up of 22.8 months and the realization of 80% of events showed that the OS HR had decreased to 0.89 (0.7-1.14) in favor of tivozanib [19]. Following the results of TIVO-3, on March 10, 2021, the FDA approved tivozanib for adult patients with relapsed or refractory metastatic advanced RCC who had previously received two or more systemic treatments [20].

Safety profile

Safety results showed that tivozanib administration generally resulted in a reduced need for dose reductions and dose interruptions compared to sorafenib. In the TIVO-1 trial, 91% of patients who received tivozanib experienced at least one treatment-emergent adverse event (AE), versus 97% in the sorafenib group [13]. The most common all-grade side effects in the tivozanib arm included hypertension (44%), diarrhea (23%), dysphonia (21%), fatigue (19%), weight loss (18%), asthenia (15%), palmar-plantar erythrodysesthesia (14%), back pain (14%), nausea (12%), stomatitis (11%), dyspnea (11%), decreased appetite (10%), and alopecia (2%) (Table 2). The most common all-grade clinical chemical abnormalities included proteinuria (72%), increased lipase (46%), increased amylase (40%), increased aspartate aminotransferase (AST) (37%), hypophosphatemia (29%), and increased alanine aminotransferase (ALT) (28%). Moreover, 41% of the patients were diagnosed with low hemoglobin, 18% with thrombocytopenia, and 11% with neutropenia. The most common grade 3 AEs included hypertension (25%), increased lipase (9%), fatigue (5%), asthenia (4%), hypophosphatemia (4%), and increased amylase (4%), while the most common grade 4 AEs included hypertension (2%), low hemoglobin (2%), and increased lipase (2%) [13].

**Table 2:** Safety of tivozanib Treatment-associated adverse effects in patients who participated in the TIVO-1 and TIVO-3 trials and received either tivozanib or sorafenib. n/a: not applicable.

	TIVO-1						TIVO-3					
	Tivozanib (n=259)			Sorafenib (n=257)			Tivozanib (n=173)			Sorafenib (n=170)		
Adverse event	All grades	Grade 3	Grade 4	All grades	Grade 3	Grade 4	All grades	Grade 3	Grade 4	All grades	Grade 3	Grade 4
Hypertension	115	66	4	88	45	1	81	35	0	46	23	0
Diarrhea	59	6	0	84	17	0	60	3	0	97	15	1
Appetite decrease	27	1	0	24	2	0	48	6	0	39	3	1
Dysphonia	55	0	0	12	0	0	41	1	0	13	0	0
Fatigue	50	14	0	41	9	0	56	6	0	36	8	0
Decreased weight	47	7	0	53	9	0	15	1	0	26	3	0
Asthenia	40	10	1	43	7	0	44	8	0	34	6	0
Palmar-plantar erythrodysesthesia	36	5	0	139	43	0	28	1	0	78	17	0
Stomatitis	29	1	0	23	2	0	35	3	0	32	4	0
Nausea	31	1	0	19	1	0	33	0	0	25	2	0
Alopecia	6	0	0	55	0	0	5	0	0	36	1	0
Rash	n/a	n/a	n/a	n/a	n/a	n/a	6	0	0	44	12	1
Vomiting	n/a	n/a	n/a	n/a	n/a	n/a	14	1	0	20	3	0
Pruritus	n/a	n/a	n/a	n/a	n/a	n/a	1	0	0	17	0	0

Compared to sorafenib, more patients in the tivozanib arm developed hypertension (44% vs. 34%), dysphonia (21% vs. 5%), back pain (14% vs. 8%), and thrombocytopenia (18% vs. 12%). On the contrary, more patients in the sorafenib group developed diarrhea (33% vs. 23%), palmar-plantar erythrodysesthesia (54% vs. 14%), alopecia (21% vs. 2%), increased AST (51% vs. 37%), increased ALT (34% vs. 28%), increased amylase (53% vs. 40%), increased lipase (64% vs. 46%), hypophosphatemia (71% vs. 29%), and low hemoglobin (49% vs. 41%). Tivozanib was associated with a lower rate of grade 3 or 4 adverse events compared to sorafenib. In addition, more patients in the sorafenib group had dose reductions (43% vs. 14%) and treatment interruptions (36% vs. 19%) due to side effects [13].

The superior safety profile of tivozanib was also validated in the TIVO-3 trial [17]. Treatment-related AEs were reported in 84% and 94% of patients in the tivozanib and sorafenib groups, respectively (Table 2). Dose interruptions occurred more frequently in the sorafenib arm (63% vs. 48%). Dose reduction occurred in 24% of the patients receiving tivozanib, compared to 38% of the patients receiving sorafenib. The tivozanib arm patients had more grade 3 or 4 hypertension (20% vs. 14%), dysphonia (1% vs. 0%), and hypothyroidism (1% vs. 0%), while patients on the sorafenib arm had more grade 3 or 4 diarrhea (0% vs. 10%), nausea (0% vs. 2%), stomatitis (0% vs. 2%), palmar-plantar erythrodysesthesia (1% vs. 10%), alopecia (0% vs. 1%), vomiting (1% vs. 2%), decreased weight (1% vs. 2%), and rash (0% vs. 8%). Grade 4 AEs were only observed in patients on the sorafenib arm (3%) [17].

Discussion

The results from the TIVO-1 and TIVO-3 trials have shown that tivozanib is another regimen with antitumor activity for patients with advanced RCC. Tivozanib had a favorable safety profile and resulted in relatively low rates of dose reductions, treatment interruptions, or discontinuations [13,17]. These data introduce tivozanib as a convenient new option for clinicians in the advanced treatment setting since it is a relatively easy medication to handle. Real-world patients who are receiving subsequent lines of therapy are frequently frail with lower performance status and cannot easily tolerate VEGFR TKIs [9]. Dose reductions and interruptions of VEGFR TKIs have been associated with worse clinical outcomes [8,21,22].

Since the recent widespread incorporation of ICIs and VEGFR TKIs in the first-line setting, alone or in doublet combinations, there is an unmet need to introduce new active regimens in subsequent lines of therapy [23]. Many of our current options for the third line and beyond, such as sunitinib and pazopanib, can potentially predispose to serious hepatotoxicity if an ICI has been previously used, due to lingering immune activation [24]. Apart from tolerability, tivozanib has shown clinical activity in this setting, since it increased the overall response rate and PFS compared to sorafenib in the TIVO-3 trial. Surprisingly, tivozanib also showed remarkable durability of responses, which is atypical for other VEGFR TKIs [17]. In the TIVO-3 trial, tivozanib resulted in superior PFS even in the subset of patients who received two previous VEGFR TKIs and even after prior axitinib administration, which is another selective VEGFR inhibitor [17,25]. However, subset analyses in the TIVO-3 trial need to be interpreted with caution because they were not the primary endpoint and because of the relatively small number of patients.

The activity of tivozanib was evident in patients with favorable IMDC risk but not in patients with poor IMDC risk [17]. This suggests that the latter tumors might be less dependent on VEGF activity. Apart from its role in tumor neo-angiogenesis and proliferation, VEGFR modulates the immune response within the tumor microenvironment [26]. Compared to other VEGFR TKIs, tivozanib exerts a stronger inhibitory action on VEGFR, which might potentially explain the durability of some responses as a result of increased immune clearance of the tumor [11]. This also suggests that a rational combination of tivozanib with an ICI might be a promising strategy against advanced RCC. A recent Ib/II clinical study showed that the combination of tivozanib/nivolumab has a tolerable safety profile with promising efficacy. The combination controlled the disease in 96% of patients and demonstrated a median PFS of 18.9 months (95% CI, 16.4 months-not reached), which was similar in treatment-naive and patients who were previously treated [27]. Ongoing studies, such as the phase 3 TiNivo-2 trial, are examining tivozanib in combination with immune checkpoint inhibitors and are expected to provide further insights on the value of such regimens in the second-line setting, following other immuno-oncology/tyrosine kinase inhibitor (IO/TKI) combinations [28].

Moreover, tivozanib did not show any survival advantage in the TIVO-3 trial, while it showed a trend toward inferior OS in the TIVO-1 trial. Most experts agree that the result of the TIVO-1 trial can be easily explained by the design of the study and the subsequent crossover of the patients. Unfortunately, the TIVO-3 study also failed to show a survival advantage of tivozanib over sorafenib [13, 17]. Although sorafenib is not the most potent VEGFR TKI against RCC, other VEGFR TKIs that are now parts of first- or second-line regimens also failed to show OS advantage over sorafenib in subsequent lines, despite the superiority in progression-free survival [29]. Direct comparisons of tivozanib with these drugs have not been conducted, although a recent retrospective study reported real-world evidence that first-line tivozanib has comparable efficacy compared to other VEGFR TKIs, particularly in patients with favorable and intermediate IMDC risk [30]. Further research is expected to provide more insight regarding the place tivozanib has among VEGFR TKIs on the sequencing of regimens in patients with mRCC.

## Conclusions

Tivozanib is a safe and tolerable VEGFR TKI that was recently added to our therapeutic armamentarium against advanced RCC, mostly in heavily pretreated patients. Rational therapeutic combinations of tivozanib are currently under investigation to determine the optimal setting in which the maximum benefit can be derived. Although it appears to be a safer and more tolerable option among other VEGFR TKIs, the efficacy benefit so far has not shown superiority, with a worrisome signal regarding short-term overall survival. Further research will determine its place in the sequencing of regimens for patients with mRCC.
